# Successful Treatment of Portal Cavernoma Cholangiopathy With Portal Vein Recanalization and Transjugular Intrahepatic Portosystemic Shunt (TIPS)

**DOI:** 10.7759/cureus.92452

**Published:** 2025-09-16

**Authors:** Michael F Kanan, Ravneet Nagra, Cynthia Papantonatos, James D Fox, Jesse Martin, Riad Salem, Keith Pereira, David Owens

**Affiliations:** 1 Department of Radiology, Saint Louis University School of Medicine, St. Louis, USA; 2 Department of Radiology, Northwestern University, Evanston, USA

**Keywords:** extrahepatic portal vein obstruction (ehpvo), portal cavernoma cholangiopathy, portal vein recanalization, portal vein thrombosis, transjugular intrahepatic portosystemic shunt (tips)

## Abstract

Portal cavernoma cholangiopathy (PCC) is a rare but serious complication of chronic extrahepatic portal vein occlusion (EHPVO), resulting from cavernous transformation of the portal vein and extrinsic compression of the bile ducts. We present a 27-year-old male with a history of portal vein thrombosis and splenomegaly who developed symptomatic PCC secondary to chronic EHPVO. The patient underwent portal vein recanalization and transjugular intrahepatic portosystemic shunt (PVR-TIPS) via trans-splenic access. Despite early stent thrombosis, successful reintervention led to restoration of portal flow and significant symptom improvement. This case highlights the clinical utility of PVR-TIPS in managing symptomatic PCC in non-cirrhotic patients and supports its consideration as a primary treatment option for decompression of the portal system and relief of biliary obstruction.

## Introduction

Portal vein occlusion poses significant morbidity and mortality to those affected, as it can lead to complications such as hypertensive gastropathy and esophageal or gastric varices, which can be fatal if left untreated. Cavernous transformation of the portal vein is a condition where numerous hypertrophied vasa vasorum vessels develop secondary to portal vein occlusion, often to bypass chronic portal vein thrombus. Portal vein cavernous transformation can be complicated by the development of portal cavernoma cholangiopathy (PCC), which is defined as an abnormality in the extrahepatic biliary system in a patient with a portal cavernoma without other cause for bile duct damage [1]. Complications of PCC include biliary stricture, chronic cholestasis, cholelithiasis, cholangitis, and abdominal pain [2]. Management of symptomatic PCC in the setting of chronic extrahepatic portal vein occlusion (EHPVO) includes biliary drainage and decompression of the portal venous system.

## Case presentation

**Figure 1:**
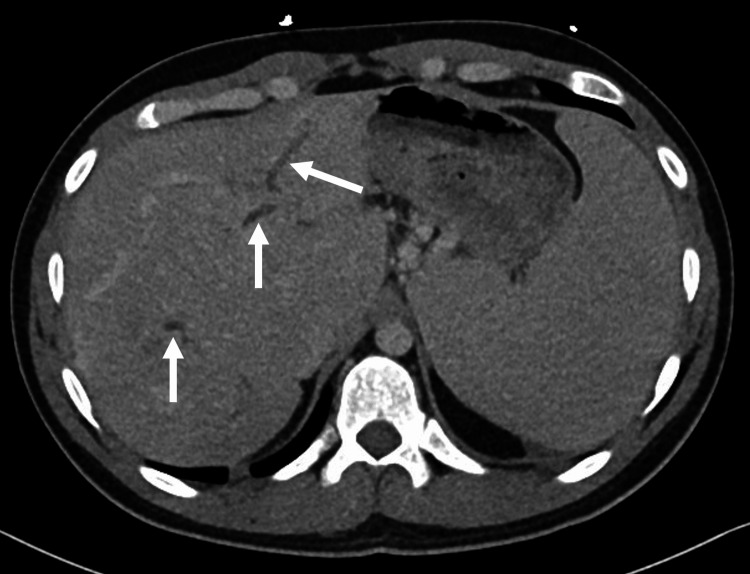
Dilated right and left hepatic lobe ducts (white arrows).

**Figure 2:**
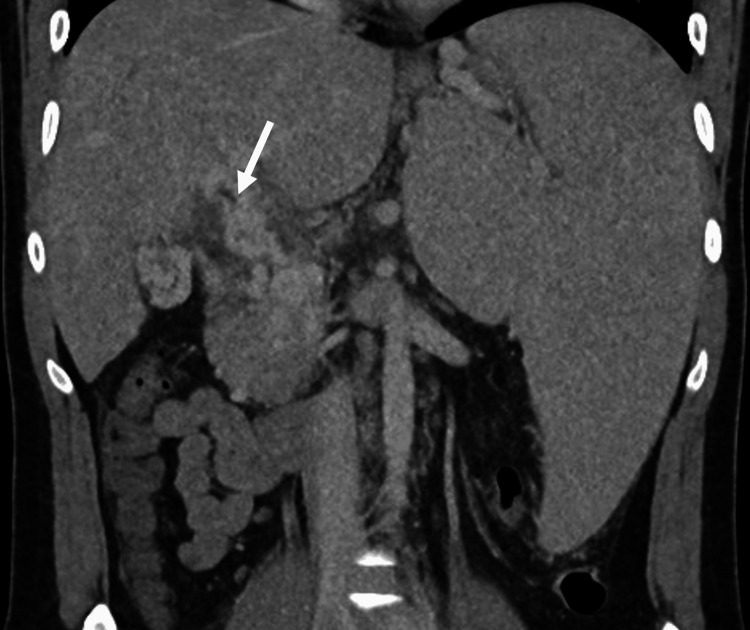
Focal narrowing of bile ducts in the cavernoma (white arrow).

**Figure 3:**
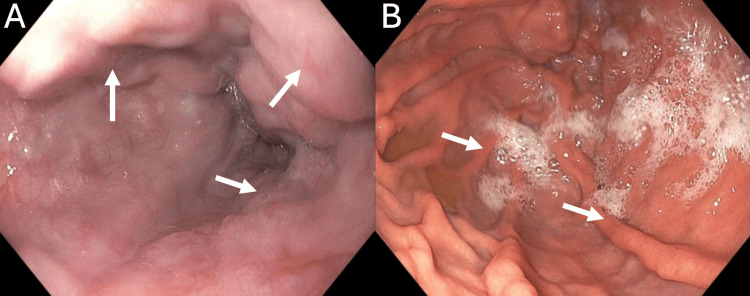
Esophageal varices and portal hypertensive gastropathy. (A) Grade II large varices (>5 mm) in the lower third of the esophagus (white arrows). (B) Mild portal hypertensive gastropathy (white arrows).

We present a case of a 27-year-old male with a past medical history notable for an unprovoked pulmonary embolism associated with thrombocytosis. His hypercoagulability workup was negative, and he was placed on anticoagulation for six months. After stopping his anticoagulation, he presented with abdominal pain, and a CT abdomen and pelvis showed portal vein thrombosis with splenomegaly and esophageal varices, requiring anticoagulation for an additional six months. Three years later, this patient presented to our institution with worsening abdominal pain, and further workup showed transaminitis with a cholestatic pattern. His CT demonstrated chronic portal vein thrombosis with cavernoma, massive splenomegaly, and intrahepatic biliary duct dilation that narrows at the level of the cavernoma, suggesting EHPVO (Figures 1, 2). Subsequent esophagogastroduodenoscopy showed large non-bleeding esophageal varices and mild portal hypertensive gastropathy (Figure 3). The patient was initially referred to interventional radiology for splenic artery embolization to treat his gastric varices. It was decided that this procedure would be unlikely to decompress the cavernoma or help with pain, so portal vein recanalization and transjugular intrahepatic portosystemic shunt (PVR-TIPS) were utilized.

**Figure 4:**
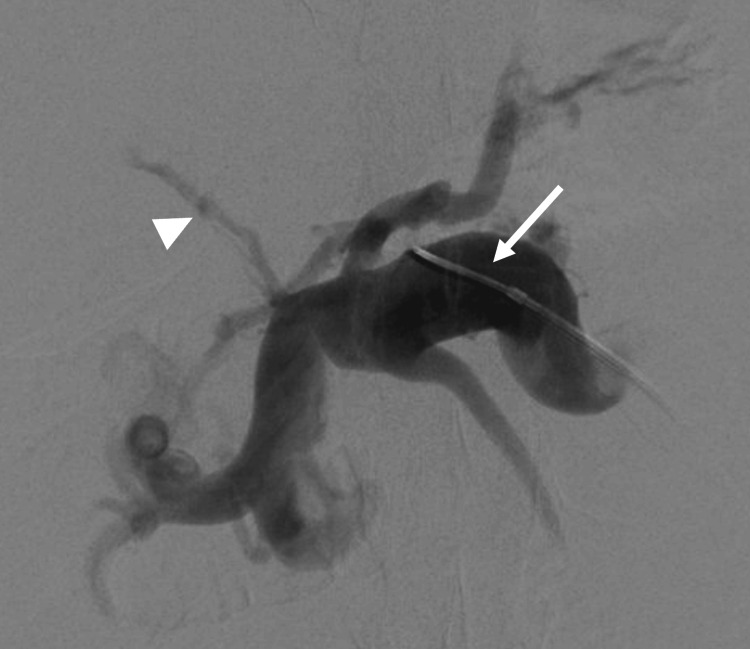
Initial portal venogram following splenic access. The white arrowhead indicates the remnant portal vein, and the white arrow indicates the trans-splenic access in the splenic vein.

**Figure 5:**
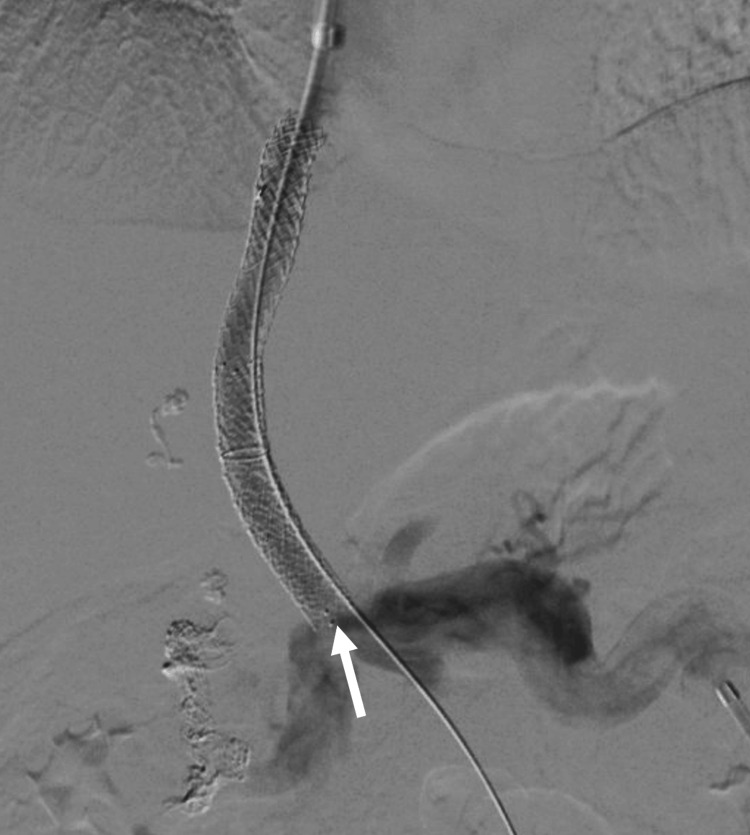
Venogram after initial TIPS placement. The white arrow indicates the recanalized portal vein at the distal end of the stent. TIPS: transjugular intrahepatic portosystemic shunt

Trans-splenic access was obtained into the portal vein with placement of a 6-French sheath. Initial portal venogram showed extensive cavernoma and a remnant cord (Figure 4). The occluded main portal vein was recanalized using a 5-French Kumpe catheter (Cook Medical, Bloomington, IN, USA) and Glidewire (Terumo Medical Corporation, Somerset, NJ, USA). A 5 mm Goose-neck snare (Medtronic, Minneapolis, MN, USA) was placed through the splenic access into the portal vein. Using a dual-sheath transjugular approach with intracardiac echocardiography (ICE) guidance and fluoroscopy, a Rosch-Uchida TIPS set (Cook Medical) was used to target the splenic vein access snare. Through-and-through access across the occluded portal vein was achieved from the internal jugular vein to splenic access. The TIPS stent was placed from the middle hepatic vein to the right portal vein and ballooned to 10 mm (Figure 5). During the procedure, the ​​patient had extensive acute thrombosis of the portal vein and TIPS refractory to large volumes of heparin. Repeat thrombectomies of the stent, portal vein, and splenic vein, and administration of anticoagulants were required to improve the thrombotic state. At the index procedure, partial cavernoma embolization was also performed to redirect flow into the TIPS stent. After the procedure, the stent was patent.

**Figure 6:**
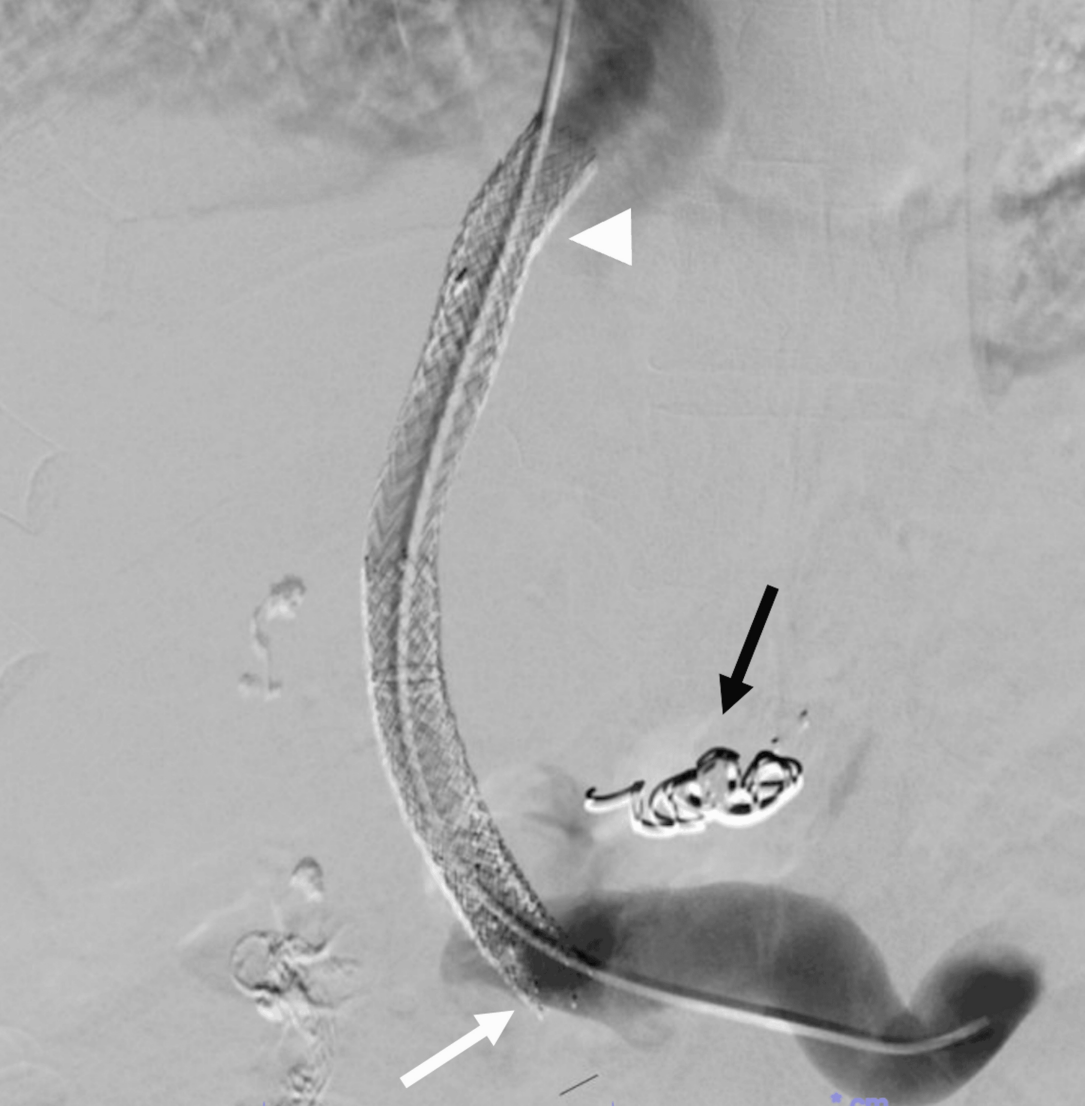
Venogram after revision and stent extension. The white arrowhead indicates the proximal stent extension, the white arrow indicates the distal stent extension, and the black arrow demonstrates a coil in the coronary varix.

**Figure 7:**
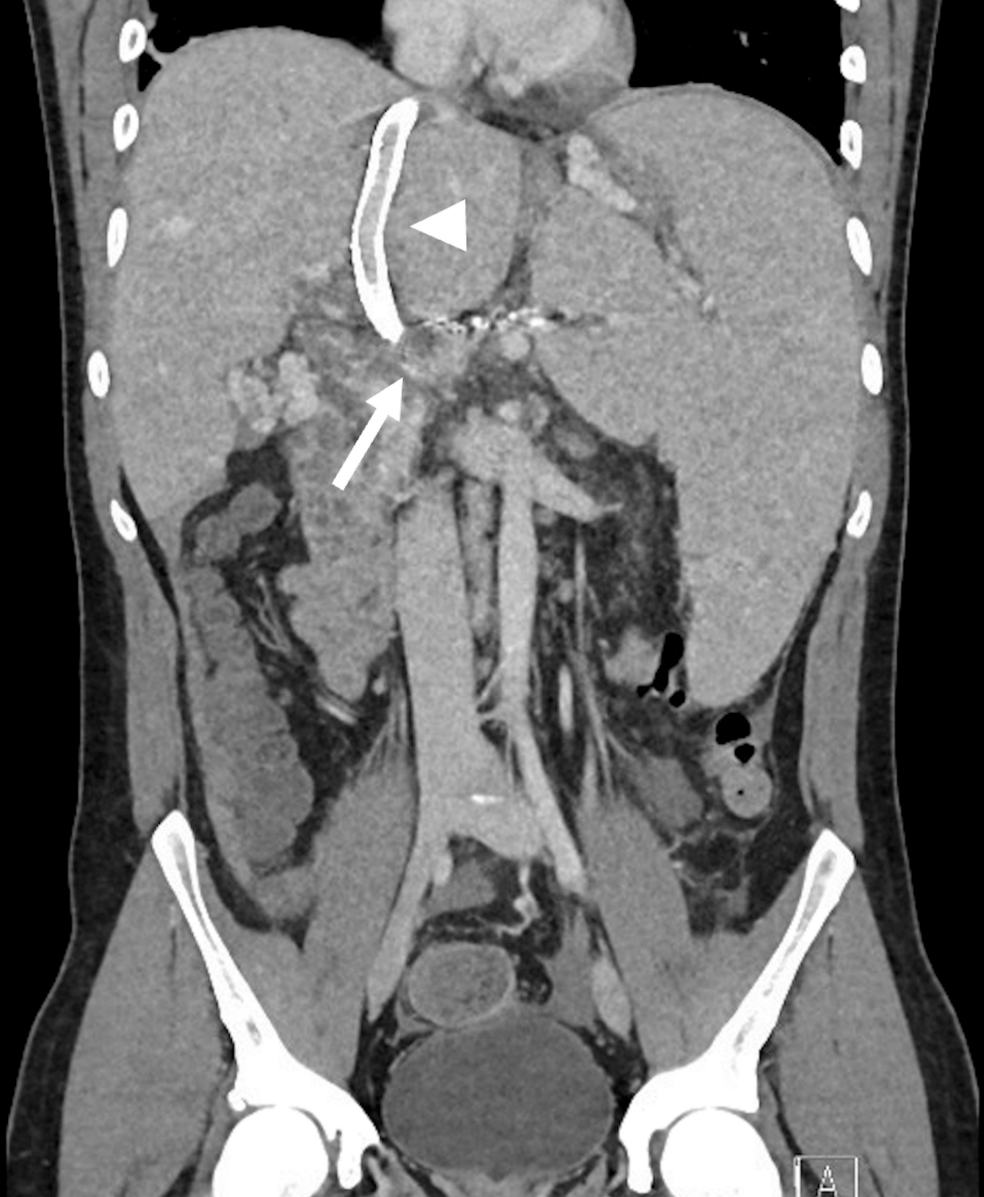
CT scan prior to discharge showing a patent TIPS (white arrowhead) and a reduced cavernoma (white arrow). TIPS: transjugular intrahepatic portosystemic shunt

Two days following the procedure, abdominal CT showed stent re-occlusion. Revision was performed with thrombectomy and extension of the proximal and distal ends of the stent with covered VBX stents (W.L. Gore & Associates, Flagstaff, AZ, USA). Prior to the completion of the second procedure, the coronary varix was coil-embolized to help maximize flow into the TIPS stent. The final portal venogram revealed brisk flow through the stent, with no visualization of the cavernoma (Figure 6). Similar findings were observed on CT scan before discharge (Figure 7) as well as on portal venogram performed one month later. On follow-up, the patient reports marked improvement in his symptoms and was placed on long-term anticoagulation.

## Discussion

EHPVO from portal vein thrombosis poses significant morbidity and mortality to those affected. EHPVO is frequently associated with the development of portosystemic collateral circulation, which can lead to cavernous transformation [3]. EHPVO is often the result of hepatic cirrhosis, although no underlying cause is detectable in around 30% of patients [4]. PCC is a known complication of portal cavernoma in the setting of EHPVO due to extrinsic mechanical obstruction of the bile ducts by collateral veins, which can present with biliary pain and cholestasis [5]. If untreated, PCC may progress to secondary biliary cirrhosis, underscoring the importance of timely treatment. While the treatment of this condition traditionally includes anticoagulation and biliary stenting, there is no established consensus regarding the treatment of PCC secondary to chronic EHPVO [5,6]. In choosing the appropriate intervention, TIPS may be a more appropriate first-line treatment for PCC, with the hope of decompressing the cavernoma and reducing biliary stenosis. By restoring hepatopetal flow, TIPS addresses the underlying hemodynamic disturbance rather than solely alleviating biliary obstruction. One study found that 87% of patients with non-cirrhotic EHPVO treated with TIPS reported subjective improvement in pain and ascites, as well as bilirubin normalization at their six-month follow-up [7]. These findings support the durability of TIPS in symptom control and metabolic improvement in this population. Furthermore, two recent cases have illustrated the effectiveness of using PVR-TIPS with trans-splenic access to treat symptomatic PCC [8,9]. This technique expands therapeutic options for patients where standard TIPS placement is technically challenging. A limitation of this case was the observed stent thrombosis following initial TIPS placement. At these authors' institutions, it has been anecdotally observed that in non-cirrhotic patients, it is often necessary to extend the stent slightly higher than one might otherwise for cirrhotic patients, which likely contributed to early stent thrombosis requiring stent extension. This case further emphasizes the potential effectiveness of treating symptomatic PCC with PVR-TIPS, while also adding to the growing body of evidence advocating for early consideration of endovascular approaches in complex portal venous pathology.

## Conclusions

This case highlights the potential effectiveness of PVR-TIPS via trans-splenic access in managing symptomatic PCC secondary to chronic EHPVO. Despite early procedural complications, successful restoration of portal flow and resolution of biliary obstruction were achieved. The patient experienced significant clinical improvement, supporting the role of PVR-TIPS as a viable therapeutic option in non-cirrhotic patients with PCC. This case adds to the growing body of evidence favoring early interventional decompression in select patients with complex portal hypertension and biliary complications.
